# Endovascular treatment of wide-neck bifurcation aneurysms using pCONUS2 HPC bridging device with single antiplatelet: A Case Series

**DOI:** 10.1097/MD.0000000000037873

**Published:** 2024-04-19

**Authors:** Jun Kiat Ho, Tze Phei Kee, Wickly Lee

**Affiliations:** a Department of Neuroradiology, National Neuroscience Institute, Singapore, Singapore

**Keywords:** antiplatelet therapy, cerebral aneurysm, endovascular treatment

## Abstract

**Rationale:**

Wide neck bifurcation aneurysms (WNBA) are technically challenging for both surgical and endovascular treatments. Endovascular treatment for WNBA often requires dual antiplatelet therapy (DAPT) post stent insertion. Novel devices such as the pCONUS2 HPC neck bridging device have an HPC coating which reduces the device thrombogenicity. This theoretically allows for use of single antiplatelet therapy (SAPT), which would be advantageous, particularly in treating ruptured aneurysms. This case series aims to evaluate the safety of SAPT regimen only post stent insertion, by presenting our center early clinical experience in using pCONUS2 HPC neck bridging device in patients that are not suitable for DAPT.

**Patient concerns:**

We report the cases of 3 patients (2 females, 1 male; range: 64–71 years old) who underwent coil embolization for WNBA using the pCONUS2 HPC device (2 unruptured WNBA, and 1 ruptured WNBA). As all 3 patients were allergic to Aspirin, they could only be started on SAPT post endovascular therapy.

**Diagnosis:**

All 3 patients were diagnosed with WNBA on angiographic studies. Patient 1 had an unruptured left middle cerebral artery aneurysm; Patient 2 had a ruptured basilar tip aneurysm; Patient 3 had an unruptured anterior communicating artery (ACOM) aneurysm.

**Interventions:**

All 3 WNBA were treated with pCONUS2 HPC neck bridging device.

**Outcomes:**

There were no immediate complications. The immediate angiographic result of aneurysm treatment in Patient 1 and Patient 2 demonstrated incomplete occlusions, with delayed complete occlusion of aneurysm in Patient 1 and growth of aneurysmal neck in Patient 2 on follow-up angiograms (range: 6–9 months). No major thrombo-embolic or hemorrhagic complications in the first 2 patients. For Patient 3, the immediate angiographic result of the treated aneurysm demonstrated complete occlusion. However, the patient readmitted 11 days post procedure with cerebral infarction, scoring 5 on the modified Rankin scale on discharge.

**Lessons:**

pCONUS2 HPC as a neck bridging device in treating WNBA has yet to be shown superior to traditional techniques and devices. The theoretical advantage of HPC coating reducing its thrombogenicity requiring only SAPT is yet to be proven safe in clinical practice.

## 1. Introduction

Wide-neck bifurcation aneurysms (WNBA) pose technical challenges for both surgical and endovascular treatment approaches due to difficulty in achieving satisfactory aneurysm occlusion while maintaining vessel patency.

Neck bridging devices are innovative apparatuses which aim to facilitate effective reconstruction of the aneurysmal neck, supporting intrasaccular coils, and preventing coil protrusion into the parent or branching vessels. The pCONUS2 is a second-generation, improved version of the first-generation pCONUS neck bridging device. It is equipped with 6 petals on the crown (compared to 4 petals in pCONUS), with more radio-opaque markers to facilitate accurate positioning within an aneurysm. Convergence of the crown to a single wire in PCONUS2 improves flexibility of the crown to articulate and accommodate various angles between the parent vessel and the aneurysm. Furthermore, the central configuration of the petals allows for good neck coverage and prevention of coil prolapse.^[1]^

The HPC surface coating on pCONUS2 is a hydrophilic surface coating that mimics the glycocalyx of epithelial cells, which aims to reduce platelet aggregation and clot formation. This HPC surface coating theoretically allows for use of single antiplatelet treatment (SAPT) as opposed to standard dual antiplatelet therapy (DAPT) following stent insertion,^[2]^ which would potentially be advantageous, especially in cases of ruptured aneurysms.

This small case series presents our center early clinical experience in using pCONUS2 HPC with SAPT and assesses its safety and efficacy.

## 2. Methods

We identified 3 patients (2 females, 1 male; range: 64–71 years old) with WNBA treated using the pCONUS2 HPC, based on our institution electronic records, from March 2021 to December 2022. All 3 patients were allergic to Aspirin. This case series fulfills our institution criteria (Singhealth centralized institutional review board) for ethics board waiver. Written informed consent was obtained from all individual participants.

### 2.1. Operative technique

All patients were treated under general anesthesia. Vascular access was established through the common femoral artery under ultrasound guidance. Systemic heparization was administered with intraprocedure activated clotting time monitoring to achieve 2 times of the baseline value or > 200 seconds. Selective cannulation of the vessels of interest was then performed, to obtain biplane and spin angiographic images with 3D reconstruction, for the purpose of treatment planning.

pCONUS2 HPC device was first deployed within the aneurysm via an 0.021” microcatheter, followed by cannulation of the aneurysm through the petals using a coiling (0.017”) microcatheter. Coil embolization of the aneurysm was then performed using Target (Stryker Neurovascular, Fremont, CA) and Kaneka i-ED coils (Kaneka medics, Kanagawa, Japan). Post embolization angiographic images were assessed for the degree of occlusion using Modified Raymond-Roy classification (MRRC) by the treating neuro-interventionalist.

The MRRC is a widely recognized system for evaluating aneurysm occlusion class. Class I is defined as complete obliteration, Class II as residual neck and Class III as residual aneurysm which is then subdivided into IIIA when the contrast opacification stays within the coil interstices of a residual aneurysm, and IIIb when the contrast opacification is outside the coil interstices, along the residual aneurysm wall. The subdivision reflected the likelihood of progression to aneurysm occlusion, as it was determined that Class IIIA was more likely than Class IIIB aneurysm to progress to either Class I (52.8% vs 6.4%, *P* < .001) or Class II (30.6% vs 8.5%, *P* < .001).^[3]^ This may be assessed on direct subtraction angiogram (DSA) or time of flight MR angiography (TOF MRA)^[4,5]^

## 3. Result

### 3.1. Patient 1

**Figure 1.**
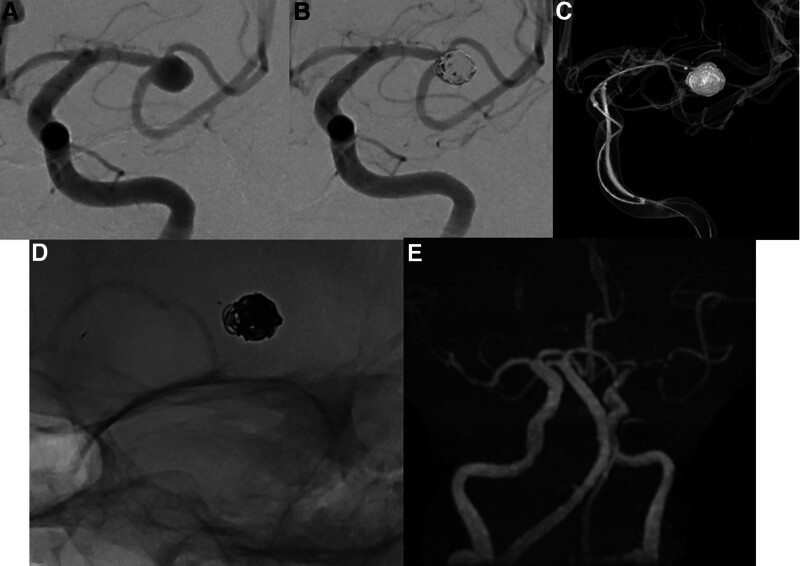
Patient 1 presented with an unruptured left MCA bifurcation aneurysm (A) treated with pCONUS 2 HPC assisted coiling (B and C). The immediate post coiling angiogram (B) showing residual contrast within the coil interstices (MRRC IIIa angiographic occlusion). 3D reconstructed image (C) and unsubtracted image (D) demonstrating the deployed pCONUS 2 HPC spanning from the left distal MCA M1-2 junction to the supraclinoid internal carotid artery (ICA) proximally, supporting the coil mass within the aneurysm sac. (E) Follow-up TOF MRA at 6 mo demonstrating complete occlusion in the treated left MCA bifurcation aneurysm. MCA = middle cerebral artery, MRRC = modified Raymond-Roy classification.

A 71-year-old female with past medical history of hypertension, hyperlipidemia, chronic kidney disease stage II, and a family history of ruptured cerebral aneurysm, was found to have an incidental left middle cerebral artery bifurcation aneurysm measuring 6.8mm (dome size) with aneurysmal neck width of 6.1mm and dome-neck ratio of 1.1 (Fig. 1A). She underwent elective coil embolization of the aneurysm, assisted with pCONUS2 HPC, achieving immediate post angiographic outcome of MRRC IIIa (Fig. 1B–D). There were no intra- or peri-procedural complications. She was started on SAPT regimen (Ticagrelor 90mg BD) 5 days prior to the procedure and continued with the same regimen post procedure. The 6 months TOF MRA follow-up showed patent stent with complete occlusion of the aneurysm with no new thrombo-embolic event (Fig. 1E). Patient remained well with no new neurological deficits.

### 3.2. Patient 2

**Figure 2.**
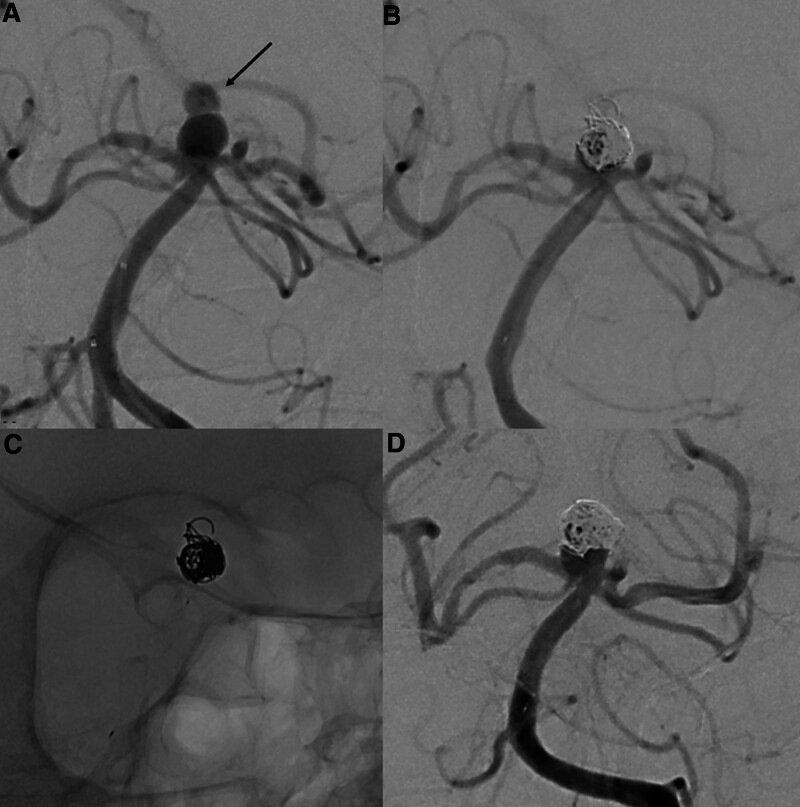
Patient 2 presented with a ruptured basilar tip aneurysm (A), with a daughter sac at its dome representing the ruptured point (arrows). The immediate angiographic image post pCONUS 2 HPC assisted coiling (B) showing residual contrast in the interstices of the coil mass—MRRC IIIB. (C) Unsubtracted image showing the deployed pCONUS 2 HPC along the basilar trunk supporting the coil mass within the aneurysm sac. (D) Follow-up angiography performed at 9 mo demonstrating interval growth of the aneurysm; the dome (the previous ruptured point) of the aneurysm remained protected. MRRC = modified Raymond-Roy classification.

A 71-year-old female with past medical history of hypertension, presented with acute subarachnoid hemorrhage from a ruptured distal basilar artery aneurysm, WFNS grade V and modified Fischer score of 4. A wide-neck basilar termination aneurysm was demonstrated on angiogram, measuring 6.2mm (dome size) with an aneurysmal neck width of 5.5 mm and a dome-neck ratio of 1.1 (Fig. 2A). She underwent endovascular coiling of the aneurysm, assisted with pCONUS2 HPC. The immediate post embolization angiographic outcome was MRRC IIIB (Fig. 2B and C). There were no intra- or peri-procedural complications. The patient was loaded with Ticagrelor 180mg pre procedure and continued on Ticagrelor 90mg BD. The 9-month DSA angiographic follow-up showed interval coil compaction with an increase in residual aneurysm size and residual flow within the coil interstices and base of the aneurysm (MRRC IIIB) (Fig. 2D). The aneurysm dome (the previous ruptured point) remain thrombosed. There was preserved flow across the parent and efferent arteries. No thrombo-embolic complications were seen and the decision was for conservative management. No follow-up imaging was available as the patient had shortly demized after from unrelated issues.

### 3.3. Patient 3

**Figure 3.**
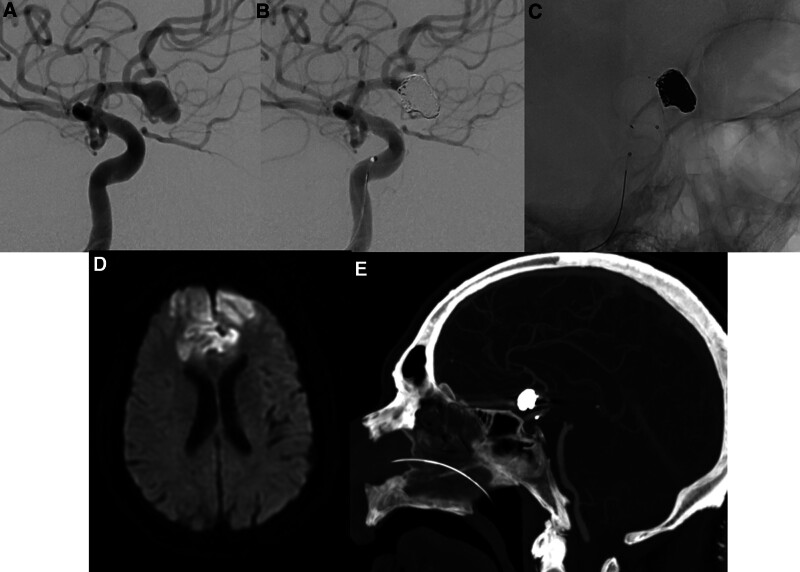
Patient 3 presented with an unruptured ACOM aneurysm (A). pCONUS 2 HPC assisted coiling was performed with complete aneurysm occlusion (MRRC I), as demonstrated on the immediate post angiographic image (B). (C) Unsubtracted image of the deployed stent and coil mass was as shown. Patient was discharged well and readmitted 10 d later for bilateral ACA territory infarcts (D). Of note, bilateral ACAs seen remained patent on the CT angiogram (E). ACA = anterior cerebral artery, ACOM = anterior communicating artery, MRRC = modified Raymond-Roy classification.

A 64-year-old male with past medical history of hypertension, hyperlipidemia, diabetes mellitus and recurrent strokes on Ticlopidine, was found to have an incidental wide-neck anterior communicating artery (ACOM) aneurysm measuring 9.0 mm (dome size) with aneurysmal neck width of 7.2 mm and dome-neck ratio of 1.2 (Fig. 3A). He underwent elective endovascular coil embolization of the ACOM aneurysm, assisted with pCONUS2 HPC, achieving immediate post embolization angiographic outcome of MRRC 1 (Fig. 3B and C). There were no intraprocedural complications. The patient was started on Ticagrelor 90mg BD 5 days prior to the procedure and continued the same regimen postprocedure. He was discharged well after the procedure the next day. 10 days later, he represented with altered mental status. MRI brain demonstrated bilateral anterior cerebral artery (ACA) territory infarcts with hemorrhagic conversion (Fig. 3D). Bilateral ACAs remained patent on multiple inpatient MR/ CT angiographic studies (Fig. 3E). He was subsequently changed to Aspirin 100mg OM, SAPT regimen, after negative drug provocation test and discussion with the neurology team. No further follow-up angiography was performed for this patient at time of the writing. He was premorbidly independent and ambulant without aids but was subsequently bedbound and dependent after this stroke, scoring 5 on the Modified Rankin Score.

### 3.4. Literature review

Literature review regarding the use of pCONUS2/ pCONUS2 HPC in WNBA identified 145 patients within 4 case series (Table 1). A total of 77.9% (113/145) achieved a satisfactory occlusion rate (defined as MRRC I or II) in the immediate angiographic evaluation. Follow-up angiographic outcomes showed a satisfactory occlusion rate of 95/127 (74.8%). The retreatment rate was 8.3% (12/144) during the 5 years follow-up period.

**Table 1:** Literature review with breakdown on the characteristics of the WNBA, immediate and delayed angiographic outcomes, retreatment rates, peri- and post procedural complications and details on the post procedural antiplatelet regimen for each case series were included.^[2,6–8]^

	No. of WNBA treated with pCONUS2/pCONUS2 HPC	No. of ruptured aneurysms	No. of unruptured aneurysms	Immediate complete occlusion rate (%)	Immediate satisfactory occlusion rate (%)	Follow-up angiographic complete + satisfactory occlusion rate (%)	Retreatment rate (%)	Peri-procedural complications (%)	Post procedural complications (%)	Ischemic stroke (%)	Hemorrhagic (%)	Others (%)	Mortality (%)	Post procedural antiplatelet regimen
Yeomans, et al, 2023^[2]^	65 (pCONUS2/pCONUS 2 HPC)	11–pCONUS2 HPC	54 - 31/54: pCONUS2 - 23/54: pCONUS2 HPC	Not available	55/65 (85)	44/60 (73)	5/65 (7.7)	6/65 (9.2)	1/65 (1.5)	1/65 (1.5)	1/65 (1.5)	5/65 (7.7)	1/65 (1.5)	Ruptured aneurysms—lifelong SAPT (6/11), DAPT for 3/6 mo before lifelong SAPT (5/11) Unruptured aneurysms—DAPT for 3/6 mo before lifelong SAPT
Morales et al, 2023^[7]^	36 pCONUS2, 20 pCONUS2 HPC	48	7	29/56 (51.8)	11/56 (19.6)	40/48 (83.3)	6/55 (10.7) - 4 retreated, 2 pending retreatment	9/56 (16.1)	0 (0)	4/56 (7.1)	0 (0)	8/56 (8.9)	0 (0)	Ruptured and unruptured aneurysms - DAPT for 6 mo followed by SAPT lifelong
Aguilar Perez, et al, 2020^[8]^	12 (pCONUS2 HPC)	12	0	1/12 (8.3)	7/12 (58.3)	4/10 (40)	0 (0)	3/12 (25)	1/12 (8.3)	1/12 (8.3)	0 (0)	3/12 (25)	0 (0)	Lifelong SAPT (ASA 100 mg BD for 3 mo followed by lifelong ASA 100 mg OM)
Aguilar Perez, et al, 2020^[6]^	12 (pCONUS2 HPC)	0	12	3/12 (25)	7/12 (58.3)	7/9 (77.8)	1/12 (8.3)	4/12 (33.3)	1/12 (8.3)	0 (0)	0 (0)	4/12 (33.3)	0 (0)	Lifelong SAPT (ASA 100 mg BD for 3 mo followed by lifelong ASA 100 mg OM)

DAPT = dual antiplatelet therapy, SAPT = single antiplatelet therapy, WNBA = wide neck bifurcation aneurysm.

The overall complication rate was 17.2% (25/145), mainly from asymptomatic stent thrombosis. The complication rate of ischemic stroke was 4.1% (6/145).

## 4. Discussion

WNBAs present a significant challenge in endovascular treatment, leading to the development of various devices and techniques over the years.

Aneurysm coiling with the assistance of balloon and stent were some of the earliest endovascular techniques in treating WNBA. Stent-assisted coiling has been shown to increase coil packing density of WNBA and subsequent angiographic improvement of aneurysm occlusion on follow-up angiogram, as compared to unassisted coiling.^[9]^ The presence of stent allows more confident coil packing of aneurysm as it reduces the risk of parent artery coil prolapse, while providing a scaffold for endothelialization across the aneurysm neck.^[9]^ DAPT is required to minimize thrombogenicity associated with stent or flow-diverter, and is hence not the preferred treatment strategy in ruptured aneurysms, unless the other treatment options are not feasible.^[10]^ Simple or balloon-assisted coiling are the preferred endovascular treatment techniques of acutely ruptured WNBA, even if the aneurysm can only be treated partially to secure the ruptured site, based on the latest AHA/ASA 2023 guideline.^[10]^

The BRANCH study demonstrated overall low complete occlusion rate (MRRC 1) of 30.6% (based on core lab assessment) at a mean follow-up of 48.8 weeks (SD ± 35.6), in wide-neck aneurysms at the middle cerebral artery bifurcation and basilar apex treated by endovascular (primary, stent- or balloon-assisted) techniques using FDA-approved devices. This study supports the ongoing need for the development and evaluation of novel endovascular devices specifically designed to treat complex intracranial aneurysms such as wide-neck aneurysm-specific bridging devices and intrasaccular flow disruption devices. Some of these devices have the theoretical advantage of being less thrombogenic and hence do not require the institution of DAPT.

The US WEB-IT study recently concluded a 5-year prospective evaluation of the efficacy of the Woven EndoBridge (WEB) aneurysm embolization system (Sequent Medical Inc). It demonstrated a last observation carried forward (LOCF) complete occlusion rate of 58.1%, and a satisfactory occlusion rate of 87.2%. Retreatment was required in 15.5% of the patients.^[11]^ The result was consistent with the 5-year results from the combined analysis of the French WEBCAST and WEBCAST-2 studies which reported similar delayed complete (51.6%) and satisfactory (77.9%) occlusion rates. Retreatment in the combined European WEBCAST studies was 11.6%.^[12]^

The pToWin study evaluated the first-generation pCONUS device, with an immediate and delayed satisfactory occlusion rate of 75.0% and 65.6% respectively. In the 12-month follow-up period, the ischemic stroke rate was 2.3%, while retreatment was required in only 4.3% of the patients.^[13]^ It is postulated that the configuration of the first-generation pCONUS device results in a gap within the aneurysm with steep angles between the parent vessel and aneurysm, causing incomplete occlusion or coil protrusion. In addition, the need for DAPT after treatment may prevent delayed thrombosis of the aneurysms. The authors postulate that the second-generation of low thrombogenic devices–PCONUS2 HPC, would better accommodate the angles between the aneurysm and the parent vessel, and allow for the use of SAPT post deployment, theoretically leading to better angiographic outcomes.^[13]^

While pCONUS2 HPC device is a relatively new device, the literature review indicated an immediate and delayed satisfactory occlusion rate of 77.9% and 74.8% respectively. This is comparable to the WEB device, although the literature also indicates that pCONUS2 has a lower retreatment rate of 8.3%.

Despite the encouraging literature review on pCONUS2, only one out of 3 patients in our study achieved satisfactory immediate angiographic occlusion. This was complicated by post procedural thrombo-embolic complications. The remaining 2 patients had residual flow within the coil interstices (mRRC IIIa) and along the aneurysm wall (mRRC IIIb) respectively, with delayed satisfactory aneurysm occlusion in the former and growth of aneurysmal neck in the latter. There were no major thrombo-embolic or hemorrhagic complications in these 2 patients. We postulate that the unsatisfactory aneurysm occlusions occurred in these 2 patients because the pCONUS2 HPC device is still unable to fully reconstruct the morphology of the aneurysm neck/ base.

Based on the literature, the pCONUS2/ pCONUS2 HPC is associated with an ischemic stroke rate of up to 8% in Aguilar Et al in their treatment of ruptured aneurysms.^[6]^ Conversely, the same author was able to demonstrate that SAPT after treatment of an unruptured WNBA is feasible without thrombo-embolic complications due to the antithrombotic properties of the pCONUS2 HPC coating.

This case series, however, suggests that standard SAPT regimen is inadequate for preventing thrombo-embolic complications. In our case series, Patient 3 suffered an ischemic stroke in bilateral ACA territories. In this patient, the ACOM aneurysm had been packed densely with coils to achieve a complete aneurysm occlusion (MRRC 1). It is postulated that the dense packing and pressure from coil mass onto the parent artery may have resulted in tiny emboli formation causing occlusion of the distal vascular territory, despite the institution of single antiplatelet. This raises the question on the need for DAPT or high-dose SAPT in patients treated with pCONUS2 HPC device.

The study has several limitations inherent with a retrospective single-arm study with a small sample size. This limits the scope of the conclusions that are drawn from the findings. In addition, analysis was performed by the treating interventionalist, and the possibility of bias should be acknowledged.

## 5. Conclusion

Based on our limited early clinical experience and literature review, the efficacy of pCONUS2 HPC as a neck bridging device in treating WNBA has yet to be shown superior to traditional techniques and devices. The theoretical advantage of HPC coating reducing its thrombogenicity requiring only SAPT has yet to be proven safe in clinical practice. Further large-scale studies are required to assess the safety and efficacy of this device.

## Author contributions

**Conceptualization:** Jun Kiat Ho, Tze Phei Kee, Wickly Lee.

**Methodology:** Jun Kiat Ho, Tze Phei Kee.

**Supervision:** Tze Phei Kee, Wickly Lee.

**Writing – original draft:** Jun Kiat Ho, Tze Phei Kee.

**Writing – review & editing:** Tze Phei Kee, Wickly Lee.
